# Modern Challenges in Type 2 Diabetes: Balancing New Medications with Multifactorial Care

**DOI:** 10.3390/biomedicines12092039

**Published:** 2024-09-07

**Authors:** Alfredo Caturano, Raffaele Galiero, Maria Rocco, Giuseppina Tagliaferri, Alessia Piacevole, Davide Nilo, Giovanni Di Lorenzo, Celestino Sardu, Erica Vetrano, Marcellino Monda, Raffaele Marfella, Luca Rinaldi, Ferdinando Carlo Sasso

**Affiliations:** 1 Department of Advanced Medical and Surgical Sciences, University of Campania Luigi Vanvitelli, 80138 Naples, Italy; alfredo.caturano@unicampania.it (A.C.); raffaele.galiero@unicampania.it (R.G.); giuseppina.tagliaferri@gmail.com (G.T.); alessia.piacevole@studenti.unicampania.it (A.P.); nilodavide@gmail.com (D.N.); giuann86@gmail.com (G.D.L.); celestino.sardu@unicampania.it (C.S.); erica.vetrano@unicampania.it (E.V.); raffaele.marfella@unicampania.it (R.M.); 2 Department of Experimental Medicine, University of Campania Luigi Vanvitelli, 80138 Naples, Italy; marcellino.monda@unicampania.it; 3 Department of Medicine and Health Sciences “Vincenzo Tiberio”, Università degli Studi del Molise, 86100 Campobasso, Italy

**Keywords:** insulin resistance, SGLT2i, GLP1-RA, tirzepatide, GIP receptor agonists, diabetes management, comprehensive care approach, pharmacological therapies

## Abstract

Type 2 diabetes mellitus (T2DM) is a prevalent chronic metabolic disorder characterized by insulin resistance and progressive beta cell dysfunction, presenting substantial global health and economic challenges. This review explores recent advancements in diabetes management, emphasizing novel pharmacological therapies and their physiological mechanisms. We highlight the transformative impact of Sodium-Glucose Cotransporter 2 inhibitor (SGLT2i) and Glucagon-Like Peptide 1 Receptor Agonist (GLP-1RA), which target specific physiological pathways to enhance glucose regulation and metabolic health. A key focus of this review is tirzepatide, a dual agonist of the glucose-dependent insulinotropic polypeptide (GIP) and GLP-1 receptors. Tirzepatide illustrates how integrating innovative mechanisms with established physiological pathways can significantly improve glycemic control and support weight management. Additionally, we explore emerging treatments such as glimins and glucokinase activators (GKAs), which offer novel strategies for enhancing insulin secretion and reducing glucose production. We also address future perspectives in diabetes management, including the potential of retatrutide as a triple receptor agonist and evolving guidelines advocating for a comprehensive, multifactorial approach to care. This approach integrates pharmacological advancements with essential lifestyle modifications—such as dietary changes, physical activity, and smoking cessation—to optimize patient outcomes. By focusing on the physiological mechanisms of these new therapies, this review underscores their role in enhancing T2DM management and highlights the importance of personalized care plans to address the complexities of the disease. This holistic perspective aims to improve patient quality of life and long-term health outcomes.

## 1. Introduction

Type 2 diabetes mellitus (T2DM) is a chronic metabolic disorder characterized by insulin resistance and progressive beta cell dysfunction, leading to hyperglycemia. It is the most common form of diabetes, accounting for about 90–95% of all diabetes cases worldwide. The prevalence of T2DM has been rising at an alarming rate, driven by factors such as population aging, urbanization, and lifestyle changes, which include unhealthy diets and physical inactivity. According to the International Diabetes Federation (IDF), as of 2021, approximately 537 million adults (20–79 years) were living with diabetes globally, a figure projected to increase to 643 million by 2030 and 783 million by 2045 [1]. This rapid rise in diabetes cases imposes a substantial burden on individuals and healthcare systems. Diabetes is associated with serious complications such as cardiovascular disease, kidney failure, neuropathy, and retinopathy, which can lead to disability and premature death [2]. In addition to the human cost, diabetes poses significant economic challenges. The global healthcare expenditure on diabetes was estimated to be USD 966 billion in 2021, an increase of 316% over the past 15 years [1]. These statistics underscore the urgent need for effective diabetes management strategies that not only control blood glucose levels but also address the broader health implications and socioeconomic impacts of the disease.

In recent years, the management of diabetes has seen significant advancements with the introduction of innovative medications that offer improved glycemic control and additional health benefits. Medications such as Sodium-Glucose Cotransporter 2 inhibitor (SGLT2i), Glucagon-Like Peptide 1 Receptor Agonist (GLP-1RA), and the novel dual glucose-dependent insulinotropic polypeptide (GIP) and GLP-1 receptor agonist, tirzepatide, have revolutionized diabetes treatment paradigms (Table 1). However, while these pharmacological advancements are crucial, managing diabetes effectively extends beyond medication alone [3,4,5]. Diabetes management, in fact, requires a multifactorial approach that addresses not only blood glucose control but also the myriad of factors that influence overall health and well-being. Pharmacological treatment alone is insufficient. Multifactorial care integrates dietary modifications, regular physical activity, weight management, and smoking cessation, which are critical in reducing the risk of complications such as cardiovascular disease, nephropathy, and neuropathy. Moreover, continuous patient education and psychological support play vital roles in enhancing treatment adherence, self-management, and quality of life. Personalized care plans must consider individual patient needs, preferences, comorbidities, and control of the entire spectrum of risk factors for cardiovascular disease, not only to prevent the onset of complications but also to enhance overall cardiovascular health. By addressing the diverse aspects of diabetes, a multifactorial care approach aims to achieve comprehensive disease management, improve patient outcomes, and reduce the burden of diabetes on individuals and healthcare systems [6].

Balancing the efficacy and benefits of new medications with comprehensive care strategies ensures that patients receive personalized, effective, and sustainable treatment, ultimately improving their overall quality of life and long-term health outcomes. This review explores the modern challenges in diabetes management by examining the latest therapeutic innovations and their integration into a holistic care framework.

## 2. Research Strategy

In preparing this review, a structured literature search was conducted to identify relevant studies on novel pharmacological therapies and their physiological mechanisms in Type 2 diabetes management. We utilized databases such as PubMed, MEDLINE, Web of Science, and Scopus, focusing on peer-reviewed articles published within the last 10 years. Our search strategy employed key terms such as “Type 2 Diabetes”, “SGLT2 inhibitors”, “GLP-1 receptor agonists”, “tirzepatide”, “Glimins”, “Glucokinase activators”, and “multifactorial care”. The inclusion criteria emphasized studies that explored the impact of these novel therapies on glycemic control, weight management, and overall metabolic health. We also prioritized studies that discussed the transition from single drug therapies to their integration within multifactorial care approaches. We included clinical trials, systematic reviews, and meta-analyses to ensure a comprehensive review of the evidence. Exclusion criteria involved studies that lacked clinical relevance and statistical power and where not written in English.

## 3. SGLT2 Inhibitors: A New Era in Diabetes Treatment

**Figure 1:**
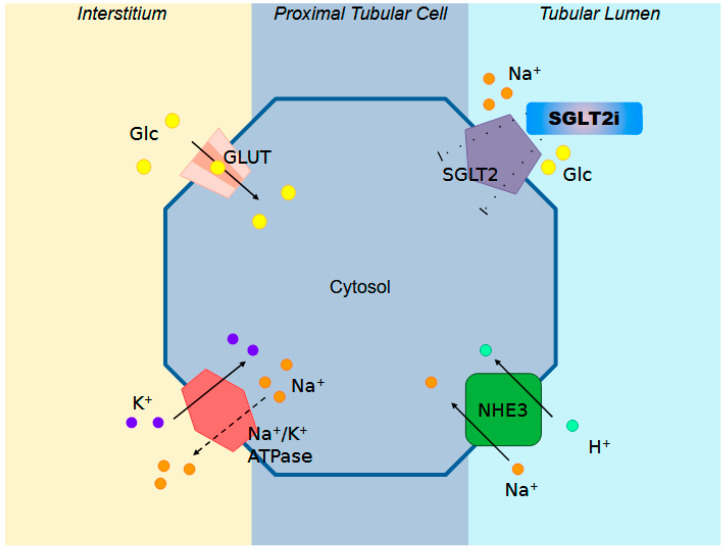
Schematic representation of the mechanism of action of Sodium-Glucose Cotransporter 2 inhibitors (SGLT2is). These inhibitors target SGLT2 transporters in the proximal renal tubules, reducing glucose reabsorption and enhancing glucose excretion in the urine, thereby lowering blood glucose levels. The figure also highlights the roles of the Na^+^/H^+^ exchanger (NHE3) and the Na+/K+ ATPase pump, which work together to maintain the sodium gradient essential for the operation of SGLT2. Additionally, the glucose transporter (GLUT) is depicted, illustrating its function in facilitating the reabsorption of glucose into the bloodstream.

Inhibitors of the SGLT2, also known as gliflozins, are a class of antihyperglycemic drugs crucial in treating T2DM [7]. Healthy kidneys filter approximately 160 g of glucose per day under euglycemic conditions; when blood glucose levels increase to the point that the filtered load exceeds this capacity, the excess is excreted in the urine [8]. Approximately 90% of filtered glucose reabsorption is mediated by SGLT2 located on the apical membrane of the S1 segment of the proximal tubule cells, while SGLT1 reabsorbs the remaining 2–3% under normoglycemic conditions [9]. SGLT2 symporters cotransport glucose and sodium in a 1:1 ratio: glucose is passively transported by GLUT1 and GLUT2 transporters at the anti-luminal site, while sodium is extruded by an active outward movement driven by ATP [10]. This sodium gradient across the apical membrane is maintained by basolateral Na^+^/K^+^-adenosine triphosphatase, which pumps out Na+ and pumps in K^+^, resulting in low intracellular Na^+^ concentration, thereby facilitating glucose reabsorption through the luminal membrane [11]. The capacity for renal glucose reabsorption is enhanced in diabetes due to SGLT2 overexpression in the proximal tubule cells (PTCs), which can be explained by their persistent exposure to high glucose levels [12]. This upregulation has been linked to the activation of angiotensin II (Ang II) AT1 receptors [13] and the transcription factor hepatocyte nuclear factor HNF-1α [14], potentially responding to basolateral hyperglycemia sensed through GLUT2 [15]. Consequently, diabetic patients have a higher threshold for urinary glucose excretion and increased glucose reabsorption compared to healthy individuals [16,17]. Under hyperglycemic conditions, increased reabsorption via SGLT1 and SGLT2 leads to a reduction in sodium concentration in the downstream tubular lumen [18]. This concentration is falsely perceived as effective hypovolemia by the macula densa at the end of the Henle loop, triggering tubulo-glomerular feedback. High sodium levels in the cells inhibit the conversion of ATP into the potent vasoconstrictor adenosine, leading to a reduction in vasodilation of the afferent arteriole, while the intrarenal activation of the renin-angiotensin-aldosterone system constricts the efferent arteriole [19,20]. The resulting increase in intraglomerular pressure induces hyperfiltration and glomerular injury with urinary albumin excretion, potentially leading to kidney damage up to overt diabetic nephropathy. The PTCs also contain the sodium/hydrogen exchanger (NHE) 3, responsible for the reabsorption of approximately two thirds of the total sodium reabsorption. NHE3 exchangers colocalize with SGLT2 symporters, and their activities are linked via the accessory membrane-associated protein 17 [21]. As a result, the increased activity of one may increase the activity of the other, explaining why SGLT2 inhibitors can block NHE3 [22,23]. Indeed, SGLT2 inhibition is associated with a marked inhibition of NHE3, even in the absence of glucose. This result can explain the significant SGLT2 inhibitor-induced natriuresis [24] (Figure 1).

The induction of glucosuria leads to improved glycemic control in all stages of T2DM, with a low risk of hypoglycemia, because they stop working when the filtered glucose load drops below 80 g per day without interfering with metabolic counter regulation [8]. This antihyperglycemic effect is influenced by specific factors. The efficacy of these drugs decreases progressively as blood glucose concentration falls [25]. Another factor is the glomerular filtration rate (GFR); the lower the GFR, the smaller the glycosuria [26]. These drugs influence glycosylated hemoglobin (HbA1c), which decreases by 7–10 mmol/mol (0.6–0.9%) compared with the placebo [27]. In addition to their main glycosuric effect, this class of drugs is characterized by pleiotropic actions, resulting in benefits for blood pressure, body weight, and particularly cardiovascular and renal protection. SGLT2 inhibition reduces body weight, initially through a diuretic effect, and subsequently by shifting substrate utilization from carbohydrates to lipids, thereby reducing body fat, including visceral and subcutaneous fat. The released free fatty acids are also used for the hepatic formation of ketone bodies, which provide additional energy substrates to improve the performance of cardiac myocytes and kidney epithelia [28,29]. By decreasing blood glucose and body weight, SGLT2 inhibitors cause a sustained improvement in beta cell function and insulin sensitivity [8]. The cardioprotective effect has been analyzed and demonstrated through several clinical trials. The clinical trials EMPAREG OUTCOME, CANVAS, and DECLARE-TIMI 58 showed that this class of drugs improved cardiovascular outcomes in T2DM patients with atherosclerotic cardiovascular disease: they significantly reduced the risk of myocardial infarction (MI), cardiovascular mortality, and all-cause mortality, although they had no effect on strokes [30,31,32]. In addition to the primary outcomes, several secondary or exploratory endpoints were collected from these trials, including those related to heart failure (HF) and kidney disease [33,34]. All gliflozins significantly reduced the risk of hospitalization for heart failure by approximately 30%, both in newly onset or recurrent HF [35]. Moreover, evidence was also reported by several real-world studies of SGLT2 is having proven to ameliorate cardiac remodeling [36,37,38,39,40]. Regarding kidney disease, several specific trials (CREDENCE, DAPA CKD, EMPA-KIDNEY) demonstrated the ability of SGLT2 inhibitors to reduce a composite of renal outcomes by 40–70%, including the doubling of serum creatinine, development of macroalbuminuria, need for dialysis and/or transplantation, and kidney death, regardless of antihyperglycemic action [41,42,43] (Table 2).

The primary and well-recognized side effects are euglycemic diabetic ketoacidosis (DKA) and urinary tract infections (UTIs) [44]. DKA has been reported with an incidence rate varying from 0.16 to 0.76 events per 1000 patient-years, with recognized risk factors including malnutrition, infectious diseases, weight loss, vomiting, or imbalanced insulin doses [45]. Moreover, to reduce the risk of euglycemic DKA, the American Diabetes Association Standards of Care recommends that SGLT2 inhibitors be discontinued 3–4 days before surgery [46]. The etiopathogenesis is still not fully clear; some authors suggest that SGLT inhibitors can stimulate lipolysis, liver ketogenesis, and a reduction in insulin production, leading to increased ketone storage and ketonemia. Additionally, it seems that the increased renal reabsorption of ketones and the hypovolemia induced by SGLT inhibitors could increase this risk [47]. The risk of UTIs is associated with glycosuria, which increases the likelihood of glucose accumulation in the urinary tract, thereby promoting bacterial growth [48]. Patients who receive proper training in regular personal hygiene can mitigate this risk. Furthermore, it appears that this effect does not extend to an increased risk of pyelonephritis or upper urinary tract infections [49]. Meta-analyses also confirmed an increased risk of genital infections, particularly among females and those with a prior history of such infections [50,51,52]. These genital infections, however, are typically non-severe and manageable without necessitating the discontinuation of treatment. An exception is Fournier’s gangrene, a rare but life-threatening condition [53,54].

There have been concerns that SGLT2is may affect mineral metabolism, potentially reducing bone density and increasing the risk of fractures [55,56]. Specifically, decreases in total hip bone mineral density (BMD) have been observed after two years of treatment with Canagliflozin. The CANVAS trial indicated a significantly higher risk of fractures overall with Canagliflozin compared to the placebo, though no significant difference in low-trauma fractures was noted. In contrast, the EMPA-REG OUTCOME and DECLARE-TIMI 58 studies did not show a significant difference in fracture risk [30,33]. Additionally, SGLT2is may predispose patients to dehydration and an increased risk of falls, warranting caution when prescribing these drugs, particularly to the elderly population [33].

**Table 2:** Summary of key clinical trials for cardiovascular and renal outcomes with diabetes medications.

Trial	Drug	Primary Outcome	Secondary Outcomes	Ref.
EMPA-REG OUTCOME	Empagliflozin	Reduced risk of cardiovascular mortality and all-cause mortality in T2DM patients	Improved heart failure outcomes, no significant effect on stroke	[30]
CANVAS	Canagliflozin	Reduced risk of myocardial infarction (MI) and cardiovascular mortality	Increased risk of fractures, reduced hospitalization for heart failure	[31]
DECLARE-TIMI 58	Dapagliflozin	Reduced risk of major cardiovascular events (MACE)	Reduced risk of hospitalization for heart failure	[32]
CREDENCE	Canagliflozin	Reduced risk of renal outcomes (e.g., doubling of serum creatinine)	Reduced progression to dialysis and kidney-related death	[41]
DAPA-CKD	Dapagliflozin	Reduced risk of renal outcomes, regardless of diabetes status	Improved cardiovascular outcomes in CKD patients	[42]
EMPA-KIDNEY	Empagliflozin	Reduced risk of progression to end-stage renal disease (ESRD)	Improved cardiovascular outcomes in kidney disease patients	[43]
LEADER	Liraglutide	Reduced MACE, including cardiovascular death, non-fatal MI, and stroke	Reduced renal mortality and macroalbuminuria	[57]
SUSTAIN-6	Semaglutide	Lowered risk of MACE in T2DM patients	Notable reductions in HbA1c and body weight	[58]
REWIND	Dulaglutide	Reduced MACE in T2DM patients with lower baseline cardiovascular risk	Reduced macroalbuminuria	[59]
HARMONY	Albiglutide	Reduced MACE in T2DM patients	–	[60]
PIONEER-6	Oral Semaglutide	Reduced MACE	–	[61]
AMPLITUDE-O	Efpeglenatide	Cardiovascular benefits similar to other GLP-1Ras	–	[62]
SELECT	Semaglutide	Reduced incidence of cardiovascular death, MI, and stroke in obese patients without T2DM	–	[63]
FLOW	Semaglutide	Nephroprotective effects, including reduced progression to ESRD	–	[64]
STEP HFpEF	Semaglutide	Improved symptoms and physical limitations in HFpEF patients	Greater weight loss, better exercise function	[65]
SURMOUNT-1	Tirzepatide	Significant weight loss in obese patients	Improved cardiovascular outcomes	[66]
SUMMIT	Tirzepatide	Ongoing trial for cardiovascular outcomes in T2DM patients	–	[67]
SURPASS-CVOT	Tirzepatide	Reduced hazard ratio for cardiovascular outcomes in T2DM patients	–	[68]

## 4. GLP-1 Receptor Agonists: Enhancing Glycemic Control and Beyond

The Glucagon-Like Peptide 1 Receptor (GLP-1R) belongs to the class B family of G protein-coupled receptors [69]. It is primarily expressed in the beta cells of the pancreas but is also found in the neurons of both the central and peripheral nervous systems, as well as various cells in the gastrointestinal tract [70]. The natural ligand for GLP-1R is the incretin hormone GLP-1, which is secreted by enteroendocrine L cells in response to food intake [71]. The binding of GLP-1 to its receptor in the pancreas initiates a signaling cascade that involves cyclic adenosine monophosphate (cAMP) and protein kinase A (PKA), leading to a series of pleiotropic effects crucial for glucose regulation [72].

Endogenous GLP-1 is rapidly degraded by the enzyme dipeptidyl peptidase 4 (DPP-4) into a less active form, which is then quickly eliminated by the kidneys [73]. Due to its short half-life—generally around 1 or 2 min in humans—direct administration of GLP-1 as a drug is limited [74]. This challenge is addressed by GLP-1RAs, which mimic the actions of GLP-1 and have been shown to be a significant advancement in the treatment of T2DM, offering benefits beyond traditional glycemic control [75]. For instance, Coskun et al. developed a GLP-1RA with a half-life of approximately five days, improving patient compliance [76].

**Figure 2:**
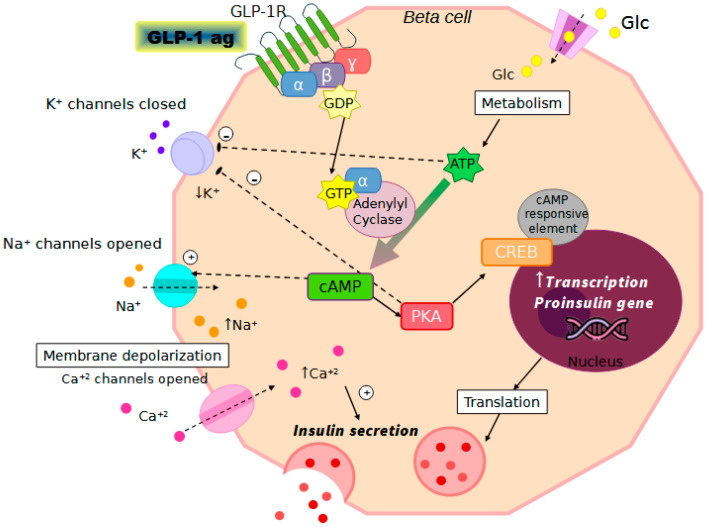
Schematic representation of the molecular pathways involved in the mechanism of action of GLP-1RAs. Upon binding to GLP-1 receptors on pancreatic beta cells, GLP-1RAs activate the cAMP/PKA signaling pathway, leading to the phosphorylation of the cAMP response element-binding protein (CREB). This activation enhances insulin gene transcription and secretion. Additionally, GLP-1RAs inhibit glucagon release from alpha cells via the same signaling pathway, thus decreasing hepatic glucose production. Colored dots represent key molecules in insulin secretion from beta cells. Orange dots (Na^+^) and red dots (Ca^2+^) illustrate ion flow crucial for membrane depolarization, which triggers the release of insulin (dark pink) and amylin (pink), aiding in glucose regulation.

GLP-1RAs enhance glycemic control through several mechanisms. Upon binding to GLP-1R on pancreatic beta cells, these agonists activate adenylyl cyclase, converting ATP into cAMP. Elevated cAMP levels activate PKA and exchange protein directly activated by cAMP (EPAC), both of which are pivotal in insulin secretion and glucose regulation [77]. PKA phosphorylates various targets, including those that regulate calcium channels, facilitating calcium entry into the cell, essential for insulin release. Additionally, PKA inhibits ATP-sensitive potassium channels, causing cell membrane depolarization and further increasing calcium influx. This rise in intracellular calcium triggers the exocytosis of insulin granules, releasing insulin into the bloodstream [78] (Figure 2).

GLP-1RAs also act on pancreatic alpha cells to inhibit glucagon secretion, thereby decreasing hepatic glucose production and contributing to lower blood glucose levels [79]. These agents slow gastric emptying, reducing the rate at which glucose is absorbed into the bloodstream postprandially. This effect is mediated through neural pathways and direct actions on the gastrointestinal tract [80]. Additionally, GLP-1RAs influence the CNS to promote satiety and reduce food intake by activating receptors in the hypothalamus and other brain regions involved in appetite regulation [81].

Beyond the primary cAMP-PKA pathway, GLP-1RAs engage several secondary signaling mechanisms. cAMP activates EPAC2, which activates proteins such as Ras-related protein 1 (RAP1) and phospholipase C (PLC). PLC generates inositol triphosphate (IP3) and diacylglycerol (DAG), promoting calcium release from intracellular stores. PKA also phosphorylates the IP3 receptor, enhancing IP3-mediated calcium release, and modulates enzymes involved in metabolic pathways, increasing glucose uptake and utilization. The combined increase in intracellular calcium and activation of downstream kinases facilitates the docking and fusion of insulin-containing vesicles with the plasma membrane, culminating in insulin release [82].

GLP-1RAs are highly effective in improving glycemic control in T2DM, lowering HbA1c levels by approximately 0.5 to 1.5% depending on the specific drug and patient characteristics [83,84,85]. Originally developed for diabetes management, GLP-1RAs like semaglutide and liraglutide have also been found to aid in weight reduction [86]. These drugs work by decreasing appetite and hunger while increasing feelings of fullness, which helps reduce overall calorie intake [87,88,89]. Beyond glycemic control, GLP-1RAs offer significant cardiovascular benefits. Several large-scale cardiovascular outcomes trials (CVOTs) have demonstrated the benefits of GLP-1 receptor agonists in both glycemic control and cardiovascular health. The LEADER trial showed that liraglutide significantly reduced major adverse cardiovascular events (MACEs), including cardiovascular death, non-fatal myocardial infarction, and non-fatal stroke, while also reducing renal mortality and macroalbuminuria in patients with T2DM [57]. Similarly, the SUSTAIN-6 trial confirmed that semaglutide lowered the risk of MACE alongside providing notable reductions in HbA1c and body weight [58]. The REWIND trial, which focused on dulaglutide, demonstrated a reduction in MACEs, even in a population with a lower baseline cardiovascular risk compared to other trials, and also showed reductions in macroalbuminuria. More recent trials continue to highlight the broad benefits of GLP-1Ras [59]. The HARMONY trial [60], with albiglutide, and the PIONEER-6 trial [61], with oral semaglutide, also demonstrated reductions in MACEs. The AMPLITUDE-O trial [62] evaluated efpeglenatide and confirmed similar cardiovascular benefits. The SELECT trial [63] and the ongoing SOUL trial [90] are further exploring the long-term cardiovascular effects of semaglutide, with a continued focus on MACE reduction. The SELECT trial showed that in patients with pre-existing cardiovascular disease and overweight or obesity but without diabetes, weekly subcutaneous semaglutide (2.4 mg) was superior to the placebo in reducing the incidence of death from cardiovascular causes, non-fatal myocardial infarction, or non-fatal stroke [61]. Additionally, newer trials such as the FLOW trial have shown the nephroprotective effects of semaglutide, including reductions in the progression to end-stage renal disease, renal mortality, and creatinine levels [64]. The STEP HFpEF trial demonstrated that in patients with heart failure with preserved ejection fraction and obesity, treatment with semaglutide (2.4 mg) led to larger reductions in symptoms and physical limitations, greater improvements in exercise function, and greater weight loss compared to the placebo [65].

Large-scale cardiovascular outcome trials (CVOTs) have consistently demonstrated that these medications can reduce major adverse cardiovascular events (MACEs), including non-fatal myocardial infarction, non-fatal stroke, and cardiovascular death [91,92,93] (Table 2). Additionally, they have been observed to lower systolic blood pressure modestly, improve lipid profiles by reducing LDL cholesterol and triglycerides, and potentially offer nephroprotective effects, such as reducing albuminuria and slowing the decline in the estimated glomerular filtration rate (eGFR).

GLP-1RAs are generally well tolerated and are not associated with an increased risk of hypoglycemia, thanks to the glucose-dependent activation of beta cells involving several intracellular mediators, such as EPAC and calcium [94]. However, they do carry some potential risks and side effects. The most common adverse effects are gastrointestinal symptoms, such as nausea, vomiting, and diarrhea, particularly when starting therapy [95]. There have been reports of acute pancreatitis associated with these medications, though a definitive causal relationship has not been established [96]. Additionally, an increased risk of gallbladder-related events, including cholelithiasis and cholecystitis, has been observed. Patients with pre-existing renal impairment should use these medications with caution, as there is potential for worsening renal function. In rodent studies, GLP-1RAs have been linked to an increased risk of thyroid C-cell tumors, but this risk has not been confirmed in humans. Injection site reactions can also occur with these drugs. Despite these potential side effects, the benefits of GLP-1RAs in managing T2DM often outweigh these risks, but healthcare providers should monitor patients closely [97].

## 5. New Frontiers: The Promise of Tirzepatide

Tirzepatide is a recently developed drug useful in the treatment of T2DM and for weight loss. This molecule shows 80% bioavailability, binds with albumin, undergoes liver metabolism through proteolytic cleavage and fatty acid β-oxidation, and is excreted via the urine and feces, with a half-life of 5 days, allowing for weekly subcutaneous administration. It is considered a long-acting molecule, with its extended activity primarily due to the addition of two residues to its lysine-linked side chain, enabling the drug to exert its benefits longer than its natural homologues [98].

**Figure 3:**
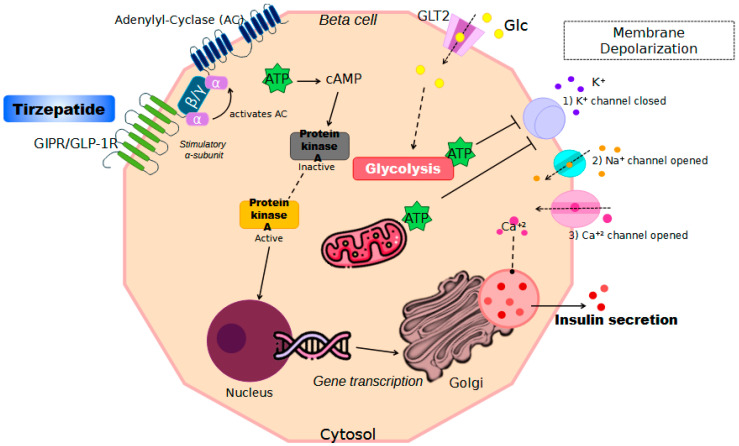
Schematic representation of the molecular pathways involved in the mechanism of action of tirzepatide in pancreatic beta cells. Tirzepatide acts on the GIPR/GLP-1R receptors, leading to the activation of adenylate cyclase (AC) and an increase in cyclic AMP (cAMP) levels. This activates protein kinase A (PKA), which in turn promotes glycolysis, ATP production, and gene transcription. The increase in ATP levels causes membrane depolarization by closing potassium (K^+^) channels, and opening sodium (Na^+^) and calcium (Ca^2+^) channels, resulting in Ca^2+^ influx. The elevated intracellular Ca^2+^ concentration stimulates insulin (dark pink) and amylin (pink) secretion.

Tirzepatide is a unimolecular dual agonist that acts as an analogue of gastric inhibitory polypeptide (GIP) and as a receptor agonist for glucagon-like peptide 1 (GLP-1). Its structure is a linear synthetic peptide comprising 39 amino acids, 19 of which are similar to those in GIP. Pharmaceutical modifications include a residue in the DPP4-binding site, making this molecule resistant to DPP4 enzymatic action; additionally, a fatty acid side chain linked to a lysine residue promotes a high-affinity bond with albumin, extending its half-life to up to 5 days. GIP and GLP-1 are involved in blood sugar homeostasis; they are secreted by cells in the human gut after food intake and regulate insulin release by pancreatic β-cells. GIP is produced by K cells in the duodenum after nutrient intake, with receptors mainly in the pancreas but also in the heart, adrenal cortex, and fat tissue. GLP-1 is secreted by L cells in the bowel, with receptors predominantly in pancreatic β-cells and, to a lesser degree, in the liver, kidneys, gastric mucosa, and brain (where it regulates satiety and food intake) [98,99] (Figure 3).

GIP is the most effective incretin-acting polypeptide in humans, and its action is triggered by increased blood glucose levels, linked with rising levels of cyclic adenosine monophosphate (cAMP). Hyperglycemia stimulates GIP secretion and promotes an increase in GIP receptor expression, enhancing cAMP levels to optimize incretin secretion. Tirzepatide’s affinity for both receptors varies; it binds more strongly to GIP receptors compared to GLP-1 receptors. This dual pathway activation significantly increases insulin secretion. Additionally, studies show that this drug improves circulating levels of adiponectin, a protein known for its role in lipid and glucose metabolism [99].

Tirzepatide’s health benefits extend to cardiovascular protection and renoprotection. Its cardiovascular benefits are closely related to enhanced GIP effectiveness, promoting anti-atherogenic effects on endothelial cells, activating endothelial nitric oxide synthase (eNOS) for vasodilation, and suppressing advanced glycation end-products (AGEs) and other atherogenic molecules. Furthermore, studies suggest potential therapeutic efficacy in lowering CD36 levels—a membrane protein involved in fatty acid import and acting as a scavenger receptor expressed mostly in abdominal fat, jejunal mucosa, and monocytes—and in suppressing inflammatory responses in macrophage foam cells [100]. A mechanism involving the reduction in triglyceride-rich lipoproteins has been postulated, contributing to the stabilization of atherosclerotic plaques. Three significant studies (SURMOUNT-1, SUMMIT [an ongoing multicentric trial], and SURPASS-CVOT) have evaluated improvements in cardiovascular outcomes in patients with obesity, heart failure, and T2DM, respectively, showing a significant reduction in the hazard ratio [66,67,68] (Table 2).

In terms of renoprotection, tirzepatide was evaluated in patients with cardiovascular disease, showing a significant reduction in kidney-worsening events, such as renal-related mortality, progression of kidney function decline to end-stage renal disease (ESRD), and new onset of macroalbuminuria, compared with treatment with insulin glargine [68]. Additionally, it is known that increased cAMP levels trigger PKA activation and inhibit oxidative stress-related kidney damage, preventing the progression of diabetic nephropathy [101]. Tirzepatide also appears to stimulate renin-secreting cells in the juxtaglomerular apparatus, enhancing natriuresis and nitric oxide levels (via decreased angiotensin II levels), thereby preventing chronic kidney injury [102].

For these reasons, tirzepatide is a promising therapeutic option for patients with T2DM and those needing significant weight loss, regardless of T2DM status. Comparative studies between standard and innovative T2DM treatments underscore the superiority of this new molecule over both placebo and active comparators (degludec [SURPASS-3], glargine [SURPASS-4], and semaglutide [SURPASS-2]), with significant reductions in HbA1c levels after 40 and 52 weeks, particularly at doses up to 15 mg/week. The same studies, following the same dosage regimen, also reported significant body weight loss [101,103,104].

Tirzepatide is generally well tolerated, with the most adverse effects being gastrointestinal symptoms, such as nausea, vomiting, and diarrhea, occurring within the first month of treatment. More serious adverse events, such as pancreatitis (3%) and cell proliferation in thyroid and pancreatic tissues, which could lead to neoplasms, are rarer [105,106,107]. The link between tirzepatide administration and endocrine cancers is currently unclear.

In conclusion, this new drug has made significant advancements in therapeutic strategies for T2DM and weight loss. Its potential to treat other metabolic conditions, such as obstructive sleep apnea syndrome (OSAS) and steatohepatitis, highlights its versatility, as demonstrated by recent studies [105,108].

## 6. Future Perspectives in Diabetes Management

The landscape of diabetes management is rapidly evolving, with promising advancements in therapies and technologies, updated guidelines, and new standards of care.

### 6.1. Glimins

Among the emerging therapies, glimins represent a new class of oral glucose-lowering drugs with mechanisms distinct from traditional medications. These molecules primarily act within the mitochondria, targeting the mitochondrial respiratory chain complex to reduce the production of reactive oxygen species (ROS) and prevent mitochondrial permeability transition pore opening, thereby protecting cells from death. They achieve this through partial inhibition of Complex I and correction of deficient Complex III activity [109].

**Figure 4:**
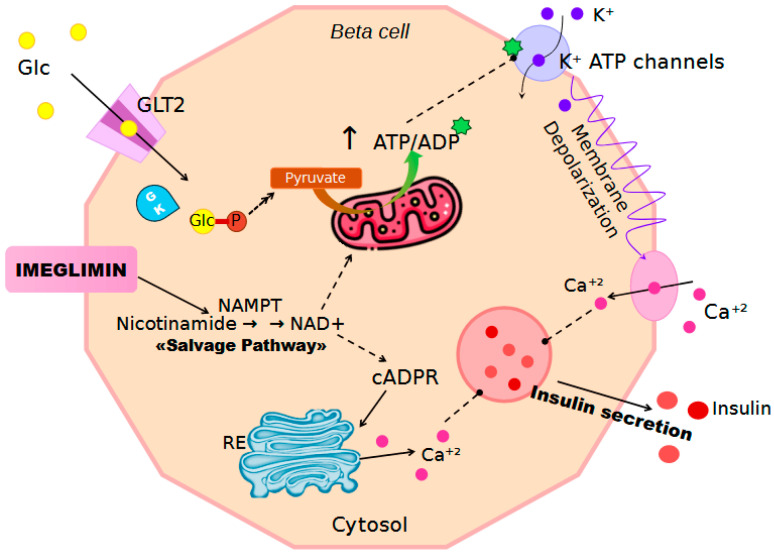
Schematic representation of the mechanism of action of Imeglimin in pancreatic β-cells. Imeglimin acts by inhibiting mitochondrial pyruvate uptake, leading to a reduction in the ATP/ADP ratio. This decrease triggers downstream effects, including the activation of nicotinamide phosphoribosyltransferase (NAMPT), which enhances the salvage pathway, converting nicotinamide into nicotinamide mononucleotide (NMN) and subsequently into nicotinamide adenine dinucleotide (NAD^+^) through the action of nicotinamide mononucleotide adenylyltransferase (NMNAT). NAD^+^ serves as a substrate for CD38, which catalyzes the formation of cyclic ADP-ribose (cADPR) and nicotinic acid adenine dinucleotide phosphate (NAADP). These molecules facilitate calcium (Ca^2+^) release from the endoplasmic reticulum (ER) and lysosomes through the ryanodine receptors (RyR) and two-pore channels (TPCs), respectively. The resulting increase in intracellular Ca^2+^ concentration contributes to membrane depolarization and promotes insulin secretion.

Glimins increase glucose-stimulated insulin secretion (GSIS), a defective process in diabetic patients, involving an NAD^+^–cyclic ADP-ribose–Ca^2+^ signaling pathway. ATP and NAD^+^ production is increased through the “salvage pathway”, with an induction of nicotinamide phosphoribosyltransferase (NAMPT) and an increase in the glucose-induced ATP pool [109] (Figure 4).

Additionally, glimins preserve β-cell mass and increase the number of insulin granules [110], while simultaneously reducing insulin resistance in insulin-sensitive tissues [111]. Glimins also exhibit a protective effect on human endothelial cells by modulating mitochondrial permeability in hyperglycemia-induced oxidative stress environments, without inhibiting mitochondrial respiration, suggesting a potential role in preventing diabetic macrovascular and microvascular complications [112].

Imeglimin, the first molecule of this new class of oral antidiabetic drugs, has shown substantial improvement in glycemic control, safety, and tolerability, even as a monotherapy [113].

### 6.2. GK Activators

Another promising therapy involves glucokinase activators (GKAs), which address the increased hepatic glucose production fundamental to the pathophysiology of T2DM. Glucokinase (GK) is the key enzyme for gluconeogenesis in hepatocytes, converting glucose into glucose-6-phosphate [114]. GK is also expressed in various other tissues, particularly in the pancreas, where it functions as a glucose sensor in beta cells, regulating insulin secretion [115]. Although there is no established correlation between pancreatic GK function and T2DM [116], GK remains inactive in the liver during fasting, forming a complex with glucokinase regulatory protein (GKRP) in the nucleus. Postprandial increases in glucose levels dissociate this complex, allowing GK to become active in the cytoplasm [117].

While mutations in the GCK gene cause maturity-onset diabetes of the young type 2 (MODY2), no GCK mutations have been clearly identified in the etiology of typical T2DM. However, Haeusler et al. discovered that in diabetic patients with high HbA1c levels (>7.0), GK expression was suppressed by more than 60%, likely due to transcriptional or post-translational effects on the enzyme [118].

**Figure 5:**
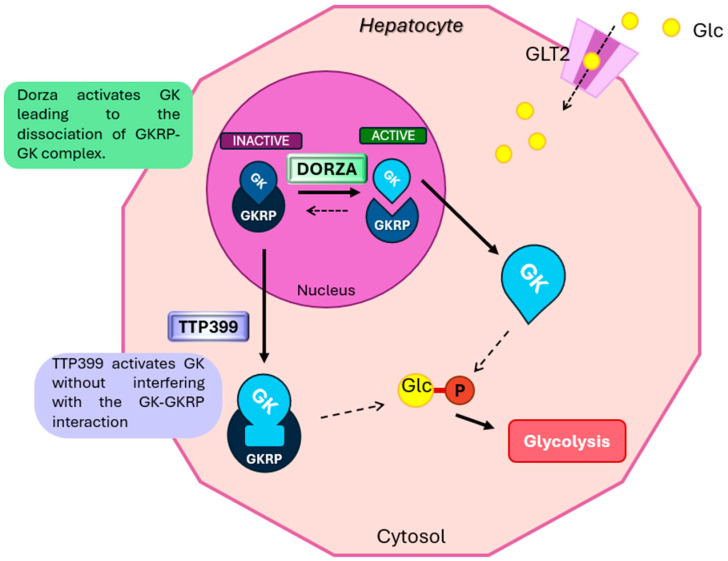
Schematic representation of the mechanism of action of glucokinase (GK) activators. The figure illustrates two distinct mechanisms by which GK activity is modulated in hepatocytes. Dorzagliatin (DORZA) promotes the dissociation of the glucokinase regulatory protein (GKRP)-GK complex within the nucleus, resulting in the release of active GK into the cytosol. This active GK facilitates the phosphorylation of glucose (Glc) to glucose-6-phosphate (Glc-6-P), a key step in glycolysis. In contrast, TTP399 activates GK directly without disrupting the GK-GKRP complex, enhancing the enzyme’s catalytic activity. Both mechanisms increase the conversion of glucose to glucose-6-phosphate, thus promoting glycolysis and enhancing glucose utilization. The figure also shows glucose entering the cell through GLUT2 transporters, emphasizing the coordinated regulation of glucose metabolism.

In this context, GKAs are a promising class of antidiabetic drugs that regulate glycemia and enhance beta cell function in T2DM patients [119]. Two recent molecules have shown great potential in terms of efficacy and safety. Dorzagliatin, also known as HMS-5552, is a dual-acting GKA that has completed two phase III trials. It targets both the liver and pancreas, working as an allosteric activator to stabilize a high-affinity conformation of the enzyme, thus increasing glucose phosphorylation activity. In the liver, Dorzagliatin activates GK, leading to the dissociation of the GK-GKRP complex. In the pancreas, the drug-activated GK inhibits insulin resistance and increases insulin sensitivity [120] (Figure 5).

TTP399, or Cadisegliatin, is a liver-specific GKA that has completed a phase II trial. Although its structural interaction with GK is still partially unclear, preclinical studies have shown that it binds to the allosteric site of GK, expanding its kinase binding cavity and increasing its catalytic activity without interfering with GK-GKRP interaction [121].

### 6.3. Retatrutide

Finally, among new drugs for diabetes treatment, retatrutide stands out as a GIP, GLP-1, and glucagon receptor triple agonist. It is a single peptide that interacts with these three different receptors. Compared to the already known tirzepatide (a GLP-1/GIP receptor agonist), retatrutide also acts on the glucagon receptor, increasing energy expenditure in mice [122].

It has been demonstrated that glucagon decreases hunger through the activation of the central nervous system via the vagus nerve. Weight loss induced by glucagon is due to increased energy expenditure, thermogenesis, and fatty acid oxidation, suggesting that this hormone impacts body weight through both feeding-dependent and -independent mechanisms [123]. In 2023, Sanyal et al. studied the effect of a 24-week treatment with retatrutide on liver fat reduction, finding that at least 80% of participants achieved >70% relative reduction in liver fat, and more than 85% achieved resolution of steatosis, defined as <5% total liver fat content. This outcome is superior to that seen with SGLT2is, dulaglutide, tirzepatide, and even semaglutide [124,125].

## 7. Evolving Guidelines and Standards of Care

In many studies, a decline in diabetes complications has been reported; various factors may be responsible for this change, but one of the most significant reasons is the improved management of risk factors [126]. According to ESC guidelines, it is recommended to screen patients with diabetes for the presence of severe target organ damage (TOD) [127,128] and for symptoms suggestive of atherosclerotic cardiovascular disease (ASCVD) [129,130]. In patients with T2DM without symptomatic ASCVD or severe TOD, it is recommended to estimate cardiovascular (CV) risk [131], considering various factors such as clinical and family history, laboratory tests, and other examinations. The 2023 ESC guidelines introduced the SCORE2-Diabetes tool to calculate the 10-year CV risk in diabetic patients, considering multiple risk factors, including the patient’s country of origin. This tool classifies 10-year CV risk into four categories: low risk (<5%), moderate risk (5–10%), high risk (10–20%), and very high risk (>20%) [131].

Once this parameter is calculated, efforts should focus on reducing CV risk through various means. Primarily, improving the patient’s lifestyle [2,132] through a balanced low-carb diet and regular exercise can induce weight loss, significantly reducing HbA1c and blood pressure [133]. Smoking cessation is also crucial, as it is associated with a 36% reduction in mortality in cardiovascular disease patients [134,135,136,137]. Kim et al. showed that smoking cessation and initiation of exercise after diabetes diagnosis are associated with a 46% reduced risk of cardiovascular disease [136]. Recent research highlights that smoking cessation, rather than reduction, is associated with reduced cardiovascular disease risk [137]. If lifestyle changes are not enough to improve glycemia and other risk factors (such as high blood pressure, lipid abnormalities, and obesity), medication should be used to reduce CV risk [138].

**Figure 6:**
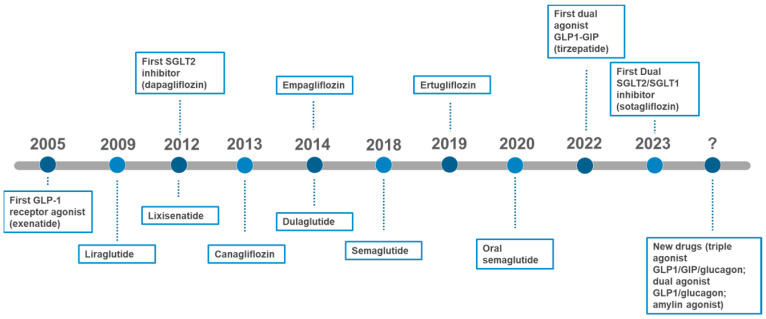
Timeline of FDA-approved drugs for the treatment of type 2 diabetes.

**Figure 7:**
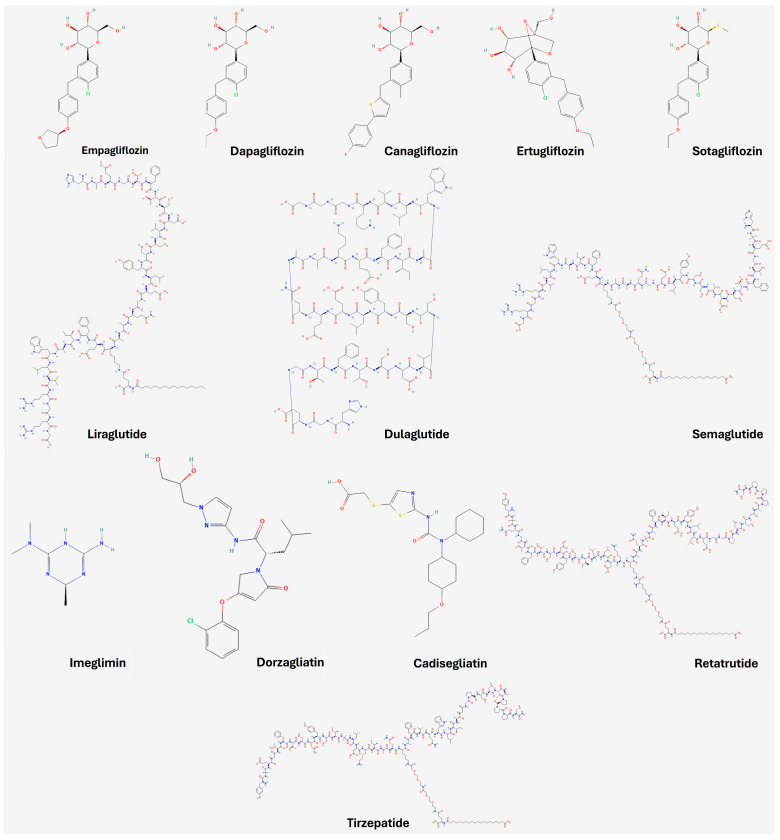
Main chemical structures of key diabetes medications [154,155,156,157,158,159,160,161,162,163,164,165,166].

Defining glycemic targets in diabetes management is complex. Tight glycemic control (HbA1c < 7%) decreases the risk of microvascular complications, but there is a U-shaped relationship between HbA1c levels and clinical outcomes, with increased mortality associated with excessively tight control. Hence, lower HbA1c is not always better [139,140,141,142,143,144,145,146,147,148,149,150]. Individualized glycemic targets should consider life expectancy, comorbidities, and diabetes duration. For patients with a short life expectancy, softer glycemic targets (HbA1c < 8.5%) may be appropriate, whereas tighter targets (HbA1c < 7%) are suitable for those with longer life expectancy, prioritizing agents with proven cardiovascular benefits and low hypoglycemic risk. It is crucial to avoid hypoglycemia, as it is associated with an increased risk of vascular events [151,152]. Figure 6 illustrates the FDA approval timeline for medications used in the treatment of type 2 diabetes. This timeline highlights key milestones in the evolution of therapeutic options, reflecting the continuous advancements in diabetes management [153]. Figure 7 complements this by presenting the chemical structures of these key medications, visually depicting the molecular innovations that underlie their therapeutic effects. As new drugs are developed and approved, they shape clinical guidelines and standards of care, offering healthcare professionals an expanding range of tools to better manage this chronic condition.

## 8. Balancing Innovation with Comprehensive Care

### 8.1. The Importance of Personalized Treatment Plans

In recent decades, scientific evidence, albeit limited, has suggested a shift from a glucocentric therapeutic vision to a multifactorial intervention approach in patients with T2DM [167]. These findings have shown that in addition to glycemic control, targeting other modifiable cardiovascular risk factors, such as blood pressure, cholesterol, lifestyle, and obesity, can prevent micro- and macrovascular complications [167,168,169].

Some hypotheses have been proposed to explain the pathophysiological mechanisms underlying the protective effects observed in subjects undergoing multifactorial interventional treatment. According to some studies, an additive protective effect is due to multifactorial intervention on cardiovascular and endothelial inflammatory damage. The effects of blood pressure-lowering treatment with RAS inhibition, the “legacy effect” on AGEs due to glucose-lowering therapy, the impact of statins on LDL and inflammatory cytokines, and the inhibition of platelet adhesion may reduce leukocyte activation and thus atherosclerosis [170,171].

The most up-to-date guidelines have highlighted the fundamental role of “tailored” therapy and the control of cardiovascular risk factors in preventing the development and progression of diabetic disease and its complications [172,173,174].

### 8.2. Role in Multifactorial Diabetes Care of New Drug Treatments

For optimal glycemic control, achieving an HbA1c of less than 7% is recommended to reduce the risk of microvascular complications while avoiding hypoglycemic events. For multifactorial intervention, guidelines suggest achieving a target systolic blood pressure of 130 mmHg and LDL-C levels of less than 100 mg/dL for individuals at moderate cardiovascular risk, less than 70 mg/dL for those at high risk, and less than 55 mg/dL for those at very high risk [172].

Currently, no single drug can address and control all cardiovascular risk factors [175]. Metformin, in the absence of contraindications, remains the first-line treatment at the time of diabetes diagnosis [176]. However, recent scientific evidence has shown that new drugs for the treatment of T2DM (GLP1-RAs and SGLT2 inhibitors) may have pleiotropic effects. Besides helping achieve glycemic targets, these drugs can prevent the progression of major cardiovascular risk factors, such as reducing blood pressure, having diuretic effects, lowering body mass index, and reducing heart filling pressures and volumes [125,176,177]. Therefore, the history and clinical characteristics of each patient should guide the best choice of tailored therapy, especially in addition to metformin [174,178,179,180]. Results from randomized placebo-controlled trials have demonstrated that in subjects with T2DM and atherosclerotic cardiovascular disease, both SGLT2 inhibitors and GLP1-RAs showed proven cardiovascular benefits, including reduced hospitalization and mortality risk. Specifically, in subjects with diabetes and heart failure with reduced ejection fraction, SGLT2 inhibitors are recommended (class I, level A evidence), with specific recommendations for empagliflozin or dapagliflozin in subjects with left ventricular ejection fraction over 40% [6]. Moreover, recent evidence has shown that chronic kidney disease (CKD) is strongly associated with the risk of developing heart failure and major adverse cardiovascular events (MACEs), especially in individuals with T2DM [180,181]. In such conditions, both SGLT2 inhibitors and GLP1-RAs are useful in reducing cardiovascular risk. For subjects with stage IV or V CKD (eGFR < 30 mL/min per 1.73 m^2^), GLP1-RAs are primarily recommended [174].

### 8.3. Addressing Patient Adherence and Lifestyle Modifications

The treatment of T2DM should always begin with lifestyle modifications. The challenge for clinicians often lies in encouraging patients to make lifestyle changes, considering their physical, social, and economic characteristics. Here again, the concept of tailored therapy is crucial in guiding clinicians toward the best choice [6].

In at least 90% of cases, T2DM is associated with overweight or obesity, which in turn often results from an unbalanced lifestyle rich in refined carbohydrates and marked by physical inactivity. Several studies have shown that a lifestyle characterized by moderate weekly physical exercise and a healthy diet can significantly reduce the risk of metabolic diseases and cardiovascular complications (RR: 0.84; 95% CI: 0.77, 0.91) [182,183]. According to guidelines, a Mediterranean diet and 180 min per week of moderate physical activity form the basis of a healthy lifestyle. However, other dietary approaches have also shown promise in reducing cardiovascular risk, though further evidence is needed to confirm these benefits [184,185,186,187,188,189].

### 8.4. Coordinating Care among Healthcare Providers

Clinicians, whether working alone or as part of a multidisciplinary team, play a central role in applying the most appropriate therapeutic strategies to address each cardiovascular risk factor. As noted earlier, some trials have demonstrated how multifactorial intervention can reduce the risk of major cardiovascular events (MACEs) and mortality [190].

The Steno-2 study was the first to address this issue. In this trial, subjects with microalbuminuria were randomly assigned to either intensive treatment or conventional therapy. The intensive treatment group aimed for stricter multifactorial treatment targets and, at the end of the study, showed a significant reduction in their risk of nephropathy (HR, 0.39; 95% CI, 0.17 to 0.87), retinopathy (HR, 0.42; 95% CI, 0.21 to 0.86), autonomic neuropathy (HR, 0.37; 95% CI, 0.18 to 0.79), stroke, and mortality (HR, 0.54; 95% CI, 0.32 to 0.89) [191,192]. In another trial, the Japan Diabetes Outcome Intervention Trial (J-DOIT3), individuals with T2DM in the intensive therapy group experienced a significant reduction in their risk of cerebrovascular events (HR, 0.42; 95% CI, 0.24 to 0.74), nephropathic events (HR, 0.68; 95% CI, 0.56 to 0.82), and retinopathic events (HR, 0.86; 95% CI, 0.74 to 1.00) [193]. More recently, the NID-2 study, a trial conducted on subjects with T2DM and high cardiovascular risk, demonstrated the efficacy of multifactorial treatment [170]. The intensive arm showed a 53% lower risk of MACEs (adjusted HR 0.47, 95% CI 0.30–0.74, *p* = 0.001) and a reduced all-cause death risk (adjusted HR 0.53, 95% CI 0.29–0.93, *p* = 0.027). Furthermore, a subsequent post hoc analysis revealed that individuals with a higher number of risk factors at target had better cardiovascular prognoses [155].

## 9. Limitations and Perspectives

This review provides a comprehensive overview of recent advancements in Type 2 diabetes management, focusing on novel pharmacological therapies. However, several limitations should be acknowledged for proper data interpretation. First, the scope of the literature covered was limited by the availability and selection of published studies, potentially introducing publication bias and restricting the inclusion of emerging but less widely reported treatments. Additionally, the heterogeneity of the included studies—varying in methodology, population, and outcome measures—may challenge the generalizability of the findings. Future research should aim to address these limitations through more systematic, interdisciplinary approaches. Moving forward, the integration of novel treatments into multifactorial care should consider individual patient variability and long-term outcomes [194]. Further exploration of emerging therapies, such as retatrutide, and the development of personalized treatment plans will be critical to advancing Type 2 diabetes care [195]. Incorporating a broader range of studies, including those that evaluate the combination of pharmacological interventions with lifestyle modifications, will be essential to optimizing patient outcomes and addressing the complexities of Type 2 diabetes management [196].

## 10. Conclusions

The management of T2DM has evolved significantly, with new therapeutic agents and a broader focus on multiple cardiovascular risk factors. This shift from a glucocentric approach has been crucial in reducing both microvascular and macrovascular complications in diabetic patients [197,198].

Medications like GLP-1 receptor agonists, SGLT2 inhibitors, tirzepatide, and glimins not only enhance glycemic control but also provide cardiovascular and renal benefits, underscoring the importance of personalized treatment plans based on individual patient characteristics [197]. Lifestyle modifications, including diet and exercise, remain foundational in T2DM management, highlighting the role of healthcare providers in coordinating comprehensive care [198]. Multidisciplinary approaches, validated in trials such as Steno-2, J-DOIT3, and NID-2, have been effective in reducing major adverse cardiovascular events (MACEs) and improving patient outcomes. These advancements emphasize the need for a holistic treatment strategy that combines emerging therapies with lifestyle interventions and thorough monitoring of cardiovascular and metabolic health [199]. As diabetes management continues to advance, a focus on individualized care and comprehensive risk factor management will be key to optimizing patient outcomes.

## Figures and Tables

**Table 1:** Overview of key diabetes medications.

Drug Class	Drug Name	Mechanism of Action	Indications	Drug Formulation	Restrictions	Adverse Effects
SGLT2i	Dapagliflozin Empagliflozin Canagliflozin Sotagliflozin Ertugliflozin	SGLT2 inhibition in the proximal tubule of the kidney, reducing glucose reabsorption	T2DM; T1DM in combination with insulin (Sotagliflozin, Dapagliflozin); HFrEF (Dapagliflozin, Empagliflozin)	Film-coated tablet	Hypersensitivity to the active substance or excipients; caution in patients at high risk of diabetic ketoacidosis	Urogenital infections, diabetic ketoacidosis, diarrhea, increased creatinine, polyuria, pollakiuria, volume depletion
GLP-1RA	Liraglutide Dulaglutide Semaglutide	GLP-1 receptor agonists: enhance insulin secretion, inhibit glucagon release, and slow gastric emptying	T2DM, obesity	Solution for injection in pre-filled pen; tablet (Semaglutide)	Hypersensitivity to the active substance or excipients	Nausea, vomiting, diarrhea, abdominal pain, decreased appetite, fatigue, local injection site reactions, cholelithiasis, gastroesophageal reflux disease, constipation, flatulence
GLP-1/GIP Receptor Agonist	Tirzepatide	Dual agonist: acts as both a GIP analogue and GLP-1 receptor agonist, enhancing insulin secretion and reducing glucagon levels	T2DM, obesity	Solution for injection in pre-filled pen	Hypersensitivity to the active substance or excipients	Nausea, vomiting, diarrhea, abdominal pain, fatigue, gastroesophageal reflux disease, constipation, flatulence, local injection site reactions, hypersensitivity reactions
Glimins	Imeglimin	Dual mechanism: improves mitochondrial function, reducing insulin resistance, and enhances insulin secretion from pancreatic beta cells	T2DM	Oral tablets	Restricted in patients with severe renal impairment or hepatic impairment	Nausea, vomiting, diarrhea, possible risk of lactic acidosis
GK Activators	Dorzagliatin	GK activator increases glucose sensitivity and enhances insulin secretion by activating glucokinase	T2DM (investigational)	Oral tablets (investigational)	Limited data available; contraindications pending further clinical trials	Hypoglycemia, gastrointestinal disturbances, potential long-term cardiovascular risks (under investigation)
GLP-1/GIP/Glucagon Receptor Agonist	Retatrutide	Triple agonist: activates GLP-1, GIP, and glucagon receptors, leading to improved glycemic control, enhanced insulin secretion, weight loss, and lipid control	Obesity, T2DM (under investigation)	Subcutaneous injection (investigational)	Contraindicated in patients with a history of medullary thyroid carcinoma or multiple endocrine neoplasia syndrome	Nausea, vomiting, diarrhea, possible risk of thyroid tumors (under investigation), pancreatitis, and gallbladder issues

## Data Availability

No dataset was generated for the publication of this article.
